# Menopause, skin and common dermatoses. Part 1: hair disorders

**DOI:** 10.1111/ced.15327

**Published:** 2022-10-28

**Authors:** Erin Kamp, Mariha Ashraf, Esra Musbahi, Claudia DeGiovanni

**Affiliations:** ^1^ Department of Dermatology University Hospitals Sussex NHS Foundation Trust Brighton UK

## Abstract

Menopause, which usually occurs between the age of 45 and 55 years, is associated with falling oestrogen levels due to ovarian follicle depletion. The impact on the cardiovascular system and bone density are well documented; however, further research required to establish the impact on the skin and hair. In this first part of a four‐part review, we examine the effect of menopause on the hair. We performed a literature review on dermatology and hair in menopause. Androgens and oestrogens are involved with regulation of the hair cycle, with a reduction in anagen hairs seen in postmenopausal women. Female pattern hair loss and frontal fibrosing alopecia have both been associated with the perimenopausal and postmenopausal states. It is clear that menopause and the change in hormone levels have an impact on the hair cycle and common hair conditions. However, further research is required, particularly to understand the therapeutic targets and role of hormonal therapy.

## Graphical abstract

Menopause is associated with falling oestrogen levels due to ovarian follicle depletion. Oestrogens and androgens are involved with regulation of the hair cycle. Both female pattern hair loss and frontal fibrosing alopecia are associated with menopause.

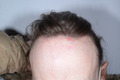

Click here for the corresponding questions to this CME article.

## Introduction

Menopause usually occurs in women between the age of 45 and 55 years.^1^ The impact on the cardiovascular system, bone density and breast tissue is well established. However, for a state in which the average woman will spend 30% of her life in, its effects on the skin and mucosal tissue require further research and understanding.^1^ In this series we will discuss the impact of menopause on hair, vulval and oral dermatology, dyspigmentation, skin ageing and common skin conditions.

## Search strategy

The Cochrane Library, National Institute for Health and Care Excellence (NICE) Evidence database and the Turning Research into Practice database were searched from 2001 to 2021. In total, 116 original research articles were found on menopause in dermatology, 13 of which related to hair in menopause. Individual searches were performed for specific queries related to our paper.

## Menopause

NICE states that diagnosis of menopause can be made without laboratory investigations in women aged > 45 years who have not menstruated for at least 12 months,^2^ excluding women using hormonal contraception or who do not have a uterus. Perimenopause, which can precede menopause by several years, is defined as irregularity of ovulation cycles and menstruation, associated with vasomotor symptoms such as night sweats and hot flushes.^1^

The mean age of menopause is 51.5 years and occurs secondary to depletion of ovarian follicles.^1^ Initially, follicle‐stimulating hormone (FSH) levels begin to rise, followed by an increase in the concentration of luteinizing hormone (LH). Progesterone and oestrogen levels then fall, with the latter being associated with irregular menstruation, vasomotor symptoms, vaginal dryness and breast atrophy.^3^

Premature menopause is diagnosed in women aged < 40 years with menopausal symptoms and laboratory tests demonstrating elevated FSH on two occasions 4–6 weeks apart.^2^ Most frequently, premature menopause is secondary to ovarian failure, the cause of which is often unknown but can be autoimmune or genetic.^4^ Oophorectomy, radiotherapy and chemotherapy can also be associated with premature menopause.^4^

**Figure 1:**
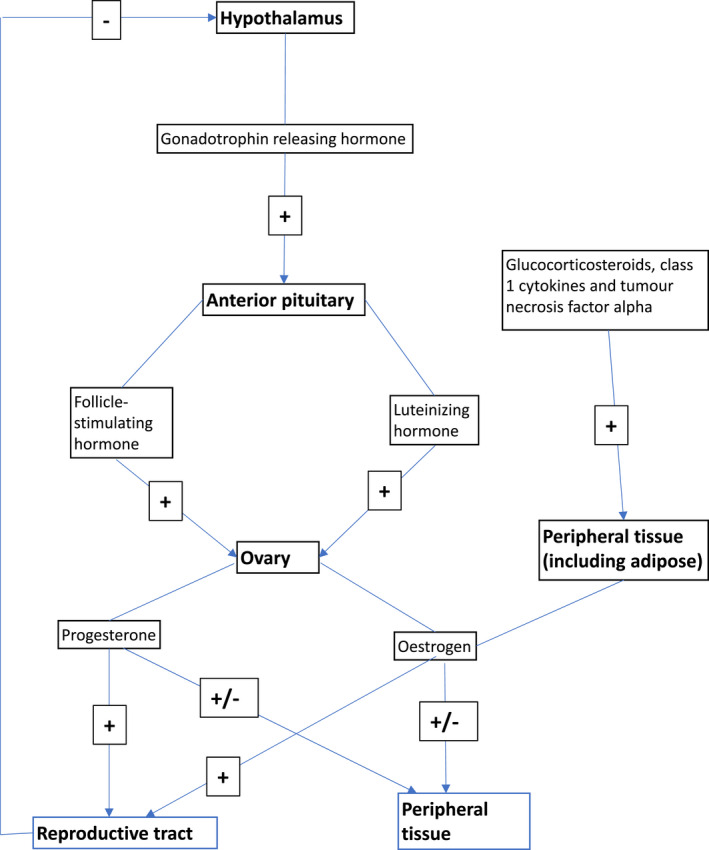
Oestrogen synthesis pathway.^1^, ^5^, ^7^

Oestrogen is synthesized from the ovaries and peripheral tissue, including the skin (Fig. 1). In premenopausal women, ovarian synthesis predominates, contrasting with the predominant synthesis from adipose tissue in postmenopausal women.^5^ The enzyme aromatase is key in oestrogen synthesis, and is present in many organs, including the skin.^5^ Oestrogen acts via two receptors, ER‐α and ER‐β; the latter is seen predominately within the skin, mucosa and hair follicles.^6^

Hormone replacement therapy (HRT) refers to the oral, transdermal or vaginal administration of oestrogen, progesterone and/or testosterone to alleviate symptoms and improve bone density. Although generally well tolerated, an increased risk of venous thromboembolism and breast cancer has been associated with HRT.^2^

## Menopause and hair

There is evidence demonstrating the role of androgens (testosterone and dihydrotestosterone), oestrogen and progesterone in the hair cycle (Table 1). Testosterone is converted to the more potent dihydrotestosterone by the enzyme 5‐α reductase and acts via intracellular receptors seen in the dermal papilla and outer root sheath of hair follicles.^8^ Androgens can influence hair follicles to change from vellus to terminal hair in the axillary, pubic region and chest. Conversely, androgens can inhibit the scalp hair follicles, a process thought to be key in the development of male pattern alopecia.^8^ Progesterone has been shown to inhibit 5‐α reductase, thereby decreasing the conversion of testosterone to dihydrotestosterone.^8^

**Table 1:** Hormonal effects on the hair follicle.^8^

Hormone	Mechanism of action	Impact on hair
Androgens	Action via intracellular androgen receptor in the dermal papilla	Face, axilla, pubic, chest hair: transformation of vellus to terminal hairs
Testosterone and dihydrotestosterone	5‐α reductase inhibitor converts testosterone to the more potent dihydrotestosterone	Scalp hair: inhibitory
Oestrogen	Binding to high‐affinity oestrogen receptors	Impact remains under investigation, but thought to prolong anagen phase and reduce telogen phase
Progesterone	Central action: inhibits LH secretion, which reduces androgen synthesis	Further research is required to understand the impact of progesterone on the hair cycle. Possible action via androgens
	Hair follicle: inhibits 5‐α reductase (reduces conversion of testosterone to dihydrotestosterone)	

LH, luteinizing hormone.

## Hair cycle abnormalities

Oestrogen is key in regulation of the hair cycle, with increased levels promoting the anagen growth phase. This is seen clinically during pregnancy and in subsequent postpartum telogen effluvium.^9^ A reduction in the proportion of anagen hairs has been demonstrated to occur in postmenopausal women, and these findings were more apparent over the frontal scalp than the occipital region.^6^

## Hair disorders after menopause

### Female pattern hair loss

Female pattern hair loss (FPHL) or androgenetic alopecia presents with thinning over the frontovertical scalp with preservation of the frontal hairline.^10^ It can be seen from early adulthood but commonly presents in menopause.^8^ The role of androgens and treatment options in male pattern alopecia are well researched; however, the role of oestrogen and androgens in FPHL is less clear.^11^ Although FPHL can be seen in hyperandrogenism, the vast majority of patients will have normal androgen levels and a reduction in oestrogen is thought to be implicated.^8^ Early‐onset and late‐onset FPHL are considered distinct conditions, with genetic factors thought to be important in both.^11^

The differential diagnoses of FPHL include chronic telogen effluvium and diffuse alopecia areata.^12^ Unless hair loss is severe or is of rapid onset, investigations are usually not required. However, free testosterone and prolactin levels are sometimes warranted if other signs of androgen excess, such as hirsutism, severe acne vulgaris and/or irregular menses are also present.^11^, ^12^

Management options are listed in Table 2. There is insufficient evidence to support the role of HRT therapy in FPHL.^11^

**Table 2:** Treatment options in female pattern hair loss.^8^, ^11^, ^12^, ^13^, ^14^, ^15^

Treatment type	Medication	Considerations
Topical	2% minoxidil solution 1–2 times/day; 5% minoxidil foam once daily	Can cause scalp irritation
Systemic	Finasterine 1 mg once daily; dutasterine 0.5–2.5 mg once daily	Suggested use of concurrent contraception if used in premenopausal women. Sexual AEs
	Cyproterone acetate 100 mg/day on days 5–15 of menstrual cycle	Studied in premenopausal women only
	Spironolactone 100–200 mg once daily	Electrolytes should be monitored 1 week after starting or after dose change, and then every 3 months
	Minoxidil 0.25–2.5 mg once daily	Caution recommended in history of cardiac disease
	Nutritional supplements (including B vitamins and zinc)	Further research needed to establish standardized dosing
Surgical	Hair transplant	Requires adequate hair density at donor site
Other	Platelet‐rich plasma	Further research required into outcomes and dosing regimens
	Low‐level laser	
	Camouflage/hairpieces/wigs	

AE, adverse effect.

The entity of senile alopecia is often considered to be on a spectrum of FPHL. Reduced hair density and weakening of the hair shaft are considered key in the diagnosis.^16^ The aetiology of both FPHL and senile alopecia is multifactoral and with limited evidence available on treatment options. The impact of the menopause and the effect of HRT need further investigation.

Some hair‐care practices are known to damage the hair shaft, and include treatments such as flat irons and colouring, used by women of all ages.^11^ With age, the hair shaft can become more fragile and less able to withstand various hair care practices, leading to damaged and broken hair.^11^

### Frontal fibrosing alopecia

Frontal fibrosing alopecia (FFA) is a scarring condition considered a variant of lichen planopilaris.^17^ It presents with progressive scarring hair loss affecting the frontal and temporal hairline. Involvement of the eyebrows is not uncommon.^10^ FFA is a disease that has only been described relatively recently, with the first cases reported in 1994.^18^ The aetiology is still under investigation, but environmental factors such as sunscreens are thought to be possible triggers.^18^ The condition has been associated with the perimenopausal state, and successful treatment with antiandrogens supports the role of hormones in the disease process.^9^, ^11^ Other treatment options are listed in Table 3.

**Table 3:** Treatment options for frontal fibrosing alopecia.^19^, ^20^

Treatment type	Medication	Considerations
Topical/intralesional	Clobetasol 0.05% cream once daily	Can exacerbate skin atrophy seen in FFA
	Tacrolimus cream 0.3–0.1% once daily	
	Minoxidil 2% solution twice daily Minoxidil 5% foam once daily	Can cause scalp irritation
	Triamcinolone 2.5 mg/mL injected every 4–6 weeks	Useful for localized disease
Systemic	Dutasteride 0.5 mg once daily	Suggested use of concurrent contraception if used in premenopausal women. Sexual AEs
	Finasteride 2.5–5 mg once daily	
	Hydroxychloroquine up to 5 mg/kg/day	Retinal screening required
	Isotretinoin 0.3 mg/kg once daily	Should be used with caution in women of childbearing age
	Pioglitazone 15 mg once daily	AEs include weight gain, bladder cancer and heart failure
	Naltrexone 3 mg once daily	Avoid use of concurrent opiates
	Tofacitinib 5 mg twice daily	Venous thromboembolism, cardiovascular events and malignancy reported
Other	Hair transplant	Risk of further scarring if FFA is not in remission or is reactivated
	Excimer or CO_2_ laser	Excimer laser thought to reduce inflammation. The mechanism of action of CO_2_ laser is unclear

AE, adverse effect; CO_2_, carbon dioxide; FFA, frontal fibrosing alopecia.

### Hirsutism

Facial hirsutism is seen in approximately 50% of postmenopausal women.^8^ Despite this, the relationship between the postmenopausal state and the development of hirsutism is yet to be elucidated. Normal androgen levels are observed in these patients, and the relative imbalance of oestrogen and testosterone has been implicated, which is supported by the role of antiandrogens in the treatment of hirsutism.^8^

Although investigations are not always required, serum testosterone levels can be helpful in excluding hyperandrogenism.^21^ If there are additional features in the clinical history and examination to suggest an underlying cause, then further investigations are often helpful. A history of irregular menstruation, acne and obesity could indicate underlying polycystic ovarian syndrome; in such cases, transvaginal ultrasonography, glucose tolerance test and lipid profile are recommended.^21^ Dexamethasone suppression test and thyroid function tests are indicated if there are features of hyperadrenocorticism or thyroid disease, respectively.^21^ In sudden‐onset and/or severe hirsutism, screening for an underlying androgen‐secreting tumour is required, and this includes transvaginal ultrasonography, computed tomography or magnetic resonance imaging of the pelvis.^16^, ^21^, ^22^

Management options include topical eflornithine as well as antiandrogens such as spironolactone. Physical epilation and laser hair removal are also commonly used.^16^

## Conclusion

It is clear from studies that there is an association between menopause and both alopecia (scarring and non‐scarring) and hirsutism. This is supported by the use of enzyme inhibition (finasteride, dutasteride) and anti‐androgens (spironolactone) as effective treatments in these conditions.

Although the exact mechanism requires further research, the association with menopause, and the dramatic changes in hormonal milieu, is evident. Despite this association, there is surprisingly little research on the role of HRT in hair conditions and further research on this is required.

In conclusion, we have discussed the hormonal changes associated with menopause and the hair conditions in which it is implicated.

**Learning points**

- Oestrogen is synthesized by the peripheral tissues, including skin, and ovaries, with the latter predominating prior to menopause.
- Androgens, oestrogen and progesterone have all been shown to affect the hair cycle.
- Increased oestrogen levels can promote the anagen phase in hair follicles.
- FPHL commonly presents in menopause; however, the role of oestrogen and progesterone is yet to be established.
- The aetiology of FFA is unclear; however, the role of antiandrogens in treatment of this condition supports the role of hormones in the disease process.
- It is postulated that relative imbalance of oestrogen and testosterone is implicated in the development of hirsutism.

## Conflict of interest

The authors declare that they have no conflict of interest.

## Funding

Funding for open access was provided by the University of Sussex as CDG is an honorary senior clinical lecturer for the university.

## Ethics statement

Ethics approval and informed consent not applicable as this was a literature review.

## Data availability

Not applicable.

## CPD questions

### Learning objective

To gain knowledge on menopause, hormonal status and associated hair disorders.

### Question 1

Which of the following statements is correct?

(a) All oestrogen is synthesized from the ovaries.
(b) Oestrogen receptor (ER)‐β is predominantly seen in the skin.
(c) Radiotherapy and chemotherapy have not been associated with premature menopause.
(d) Luteinizing hormone (LH) levels are the first to increase in perimenopause.
(e) Blood tests are required to diagnose menopause.

### Question 2

Which of the following statements about female pattern hair loss (FPHL) is true?

(a) FPHL presents with thinning over the vertex and occipital scalp.
(b) FPHL is never seen before menopause.
(c) The majority of patients with FPHL will have hyperandrogenism.
(d) Genetic factors are thought to be implicated in FPHL.
(e) The differential diagnoses include anagen effluvium.

### Question 3

Which of the following statements about frontal fibrosing alopecia (FFA) are correct?

(a) FFA affects the scalp only.
(b) Topical treatment such as corticosteroids and minoxidil do not play a role in disease management.
(c) Environmental triggers are not implicated in FFA.
(d) FFA is associated with menopause and perimenopause.
(e) Hair transplantation is recommended early in the disease.

### Question 4

Which of the following statements about hirsutism is correct?

(a) Hirsutism is seen in only a minority of postmenopausal women.
(b) It is thought that an imbalance between progesterone and oestrogen is implicated in the pathogenesis.
(c) Sudden‐onset and/or severe hirsutism are normal and do not require any further investigations.
(d) Thyroid function tests are not part of the investigations for hirsutism.
(e) Pelvic imaging can be helpful in excluding an androgen‐secreting tumour if suspected clinically.

### Question 5

Which of the following statements about hormones and the hair cycle is correct?

(a) Dihydrotestosterone is converted to testosterone by the enzyme 5‐α reductase.
(b) Androgens can influence hair follicles to change from vellus to terminal hair on the scalp.
(c) Progesterone has been shown to inhibit 5‐α reductase.
(d) Oestrogen is thought to prolong the telogen phase.
(e) Progesterone can inhibit secretion of follicle‐stimulating hormone (FSH).

## Instructions for answering questions

This learning activity is freely available online at http://www.wileyhealthlearning.com/ced

Users are encouraged to

- Read the article in print or online, paying particular attention to the learning points and any author conflict of interest disclosures.
- Reflect on the article.
- Register or login online at http://www.wileyhealthlearning.com/ced and answer the CPD questions.
- Complete the required evaluation component of the activity.

Once the test is passed, you will receive a certificate and the learning activity can be added to your RCP CPD diary as a self‐certified entry.

This activity will be available for CPD credit for 2 years following its publication date. At that time, it will be reviewed and potentially updated and extended for an additional period.
